# Multiscale Entropy Approaches and Their Applications

**DOI:** 10.3390/e22060644

**Published:** 2020-06-10

**Authors:** Anne Humeau-Heurtier

**Affiliations:** LARIS—Laboratoire Angevin de Recherche en Ingénierie des Systèmes, University of Angers, 49035 Angers, France; anne.humeau@univ-angers.fr

**Keywords:** multiscale entropy, multivariate data, entropy

## 1. Introduction

Multiscale entropy (MSE) measures have been proposed from the beginning of the 2000s to evaluate the complexity of time series, by taking into account the multiple time scales in physical systems. Since then, these approaches have received a great deal of attention and have been used in a large range of applications. Multivariate approaches have also been developed.

The algorithms for a MSE approach are composed of two main steps: (i) a coarse-graining procedure to represent the system’s dynamics on different scales; and (ii) the entropy computation for the original signal and for the coarse-grained time series to evaluate the irregularity for each scale. Moreover, different entropy measures have been associated with the coarse-graining approach, each one having its advantages and drawbacks: approximate entropy, sample entropy, permutation entropy, fuzzy entropy, distribution entropy, dispersion entropy, etc. 

In this Special Issue, we gathered 24 papers focusing on either the theory or applications of MSE approaches. These papers can be divided into two groups: papers that propose either new developments on entropy-based measures or improve the understanding of existing ones (nine papers); and papers that propose new applications of existing entropy-based measures (14 papers), as described below. Moreover, one paper proposes a review on cross-entropy methods and their multiscale approaches [1].

## 2. New Developments in Entropy-Based Measures

Lee et al. proposed a multiscale distribution entropy based on a moving averaging multiscale process and distribution entropy to study short-term heart rate variability (HRV) [2]. The authors show that the new entropy-based measure outperforms MSE and multiscale permutation entropy as it is insensitive to the length of signals. The new measure shows a decrease in the complexity of HRV with aging and for congestive heart failure patients.

Zhao et al. proposed the multiscale entropy difference (MED) to assess the predictability of nonlinear financial time series on several time scales [3]. MED quantifies the contributions of the past values by reducing the uncertainty of the forthcoming values in signals on several time scales. The algorithm has been validated on simulated data and then applied to the analysis of Chinese stock markets. 

Cheng et al. proposed a method based on multimodal multiscale dispersion entropy for the biometric characterization of heart sounds [4]. The work relies on the use of the improved complete ensemble empirical mode decomposition with adaptive noise (ICEEMDAN) and refined composite multiscale dispersion entropy. The authors show that the proposed method is effective for heart sound biometric recognition. 

Dong et al. proposed a method, KeepSampEn, to minimize the error due to missing values in sample entropy calculation [5]. For this purpose, they modified the computation process but not the data. The results reveal that KeepSampEn shows a consistent lower average percentage error than other methods as skipping the missing values, linear interpolation and bootstrapping. 

Tiwari et al. investigated the multiscale features of the mental workload for ambulant users [6]. Features that outperform benchmark ones are proposed and they exhibit complementarity when used in combination. Thus, the authors reported that composite coarse-graining via a new second moment moving average scaling method, combined with the modified permutation entropy method, outperforms other combinations. 

From a Taylor series expansion, Dávalos et al. developed an explicit expression for the multiscale permutation entropy (MPE) estimator’s variance as a function of the time scale and ordinal pattern distribution [7]. They also determined the Cramér–Rao lower bound of the MPE. The results show that MPE variance is related to the MPE measurement and increases linearly with time scale, but not when the MPE measure reaches its maximum value. Moreover, for short time scales compared to the signal length, the MPE variance resembles the MPE Cramér–Rao lower bound. 

Bajic et al. proposed a method that enables an application of MSE to an arbitrary number of signals [8]. The authors also wanted to test whether their method recognizes the changes of the dependency level (coupling strength, level of interaction) of joint multivariate signals in different biomedical experiments. For this purpose, they use the copula density to determine the coupling strength. Moreover, the authors apply the composite MSE to the systolic blood pressure, the pulse interval, and the body temperature of rats exposed to different ambient temperatures. 

Azami et al. introduced the multivariate multiscale dispersion entropy (mvMDE) to quantify the complexity of multivariate time series [9]. When applied to different kinds of signals, the results show that mvMDE has some advantages over multivariate multiscale entropy (mvMSE) and multivariate multiscale fuzzy entropy (mvMFE). 

Martins et al. introduced a new method to assess the complexity of multivariate time series [10]. This new method takes into account the presence of short-term dynamics and long-range correlations and uses vector autoregressive fractionally integrated (VARFI) models. This leads to a linear parametric representation of the vector’s stochastic processes. Then, an analytical formulation is obtained to derive the MSE measures. The authors tested this new approach on cardiovascular and respiratory signals to assess the complexity of the heart period, the systolic arterial pressure, and the respiration variability in different physiological conditions. The results show that, by taking into account long-range correlations, the method proposed by the authors overcomes the existing ones as it captures significant variations in the complexity that are not observed with standard existing methods.

## 3. Applications of Existing Entropy-Based Measures

In this Special Issue, 14 papers propose to use existing entropy-based measures for different kinds of applications, as mentioned below.

Harezlak et al. studied eye movement signal characteristics [11]. For this purpose, the authors used several methods: approximate entropy, fuzzy entropy, and the largest Lyapunov exponent. For these three methods, multilevel maps are defined. The results show better accuracy for saccadic latency and saccade, than previous studies using eye movement dynamics.

Liau et al. evaluated the changes in the complexity of the center of pressure (COP) during walking at different speeds and for different durations [12]. For this purpose, the MSE was used. The authors show that both the walking speed and walking duration factors significantly affect the complexity of COP. 

Based on ensemble empirical mode decomposition (EEMD) and MSE and using an accelerometer, Nurwulan et al. proposed a measure, the postural stability index (PSI), to distinguish different stability states in healthy subjects [13]. PSI is able to discriminate between normal walking and walking with obstacles in healthy subjects.

McDonough et al. were interested by post-encoding memory consolidation mechanisms in a sample of young, middle-aged and older adults [14]. For this purpose, they tested a novel measure of information processing, network complexity and studied if it was sensitive to these post-encoding mechanisms. Network complexity was determined by assessing the irregularity of brain signals within a network over time. This was performed through MSE. The results show that network complexity is sensitive to post-encoding consolidation mechanisms that enhance memory performance. 

Menon and Krishnamurthy mapped neuronal and functional complexities from the MSE of resting-state functional magnetic resonance imaging (rfMRI) blood oxygen-level dependent (BOLD) signals and BOLD phase coherence connectivity [15]. 

De Wel et al. proposed a novel unsupervised method to discriminate quiet sleep from non-quiet sleep in preterm infants, from the decomposition of a multiscale entropy tensor [16]. This was performed according to the difference in the electroencephalography (EEG) complexity between the neonatal sleep stages. 

Jelinek et al. investigated the efficacy of applying multiscale Renyi entropy on heart rate variability (HRV) to obtain information on the sign, magnitude, and acceleration of the signals with time [17]. The results show that their quantification using multiscale Renyi entropy leads to statistically significant differences between the disease classes of normal, early cardiac autonomic neuropathy (CAN), and definite CAN. 

El-Yaagoubi et al. studied the dynamics, the consistency and the robustness of MSE, multiscale time irreversibility (MTI), and multifractal spectrum in HRV characterization in long-term scenarios (7 days) [18]. The results show that congestive heart failure (CHF) and atrial fibrillation (AF) populations show significant differences at long-term and very long-term scales (thus, MSE is higher for AF while MTI is lower for AF). 

For an early Alzheimer’s disease (AD) diagnosis, Perpetuini et al. used sample entropy and the MSE of functional near infrared spectroscopy (fNIRS) in the frontal cortex of early AD and healthy controls during three tests that were used to assess visuo-spatial and short-term-memory abilities [19]. A multivariate analysis revealed promising results (good specificity and sensitivity) in the capabilities of fNIRS and complexity for an early diagnosis. 

Keshmiri et al. studied the effect of the physical embodiment on older people’s prefrontal cortex (PFC) activity when they are listening to stories [20]. For this purpose, they used MSE. Their results show that, in older people, physical embodiment leads to a significant increase of MSE for PFC activity. Moreover, this increase reflects the perceived feeling of fatigue.

Xu et al. used the short-time series MSE (sMSE) to study the complexities and temporal correlations of Wikipedia page views of four selected topics [21]. The goal was to understand the complexity of human website searching activities. The results show that sMSE is useful to analyze the temporal variations of the complexity of page view data for some topics. Nevertheless, the regular variations of sample entropy cannot be accepted as is when different topics are compared. 

Lin et al. developed an entropy-based structural health monitoring system to solve the problem of unstable entropy values observed when multiscale cross-sample entropy was used to assess damage in laboratory-scale structure [22]. The results could be interesting for long-term monitoring. 

Ge et al. proposed a bearing fault diagnosis technique using the local robust principal component analysis (to remove background noise: it decomposed the signal trajectory matrix into multiple low-rank matrices) and multiscale permutation entropy that identified the low-rank matrices corresponding to the bearing’s fault feature [23]. The latter matrices are then combined into a one-dimensional signal and represents the extracted fault feature component. 

Shang et al. used variational mode decomposition and multiscale dispersion entropy to propose a novel feature extraction method for partial discharge fault analysis [24]. Moreover, a hypersphere multiclass support vector machine was used for partial discharge pattern recognition. 

Let us now hope that these papers will bring other interesting applications and lead to new ideas to further improve the study of the irregularity and complexity of data (1D, 2D, *n*-D).
